# Hummingbirds use wing inertial effects to improve manoeuvrability

**DOI:** 10.1098/rsif.2023.0229

**Published:** 2023-10-04

**Authors:** Mohammad Nasirul Haque, Bo Cheng, Bret W. Tobalske, Haoxiang Luo

**Affiliations:** ^1^ Department of Mechanical Engineering, Vanderbilt University, Nashville, TN 37235, USA; ^2^ Department of Mechanical Engineering, Pennsylvania State University, University Park, PA 16802, USA; ^3^ Field Research Station at Fort Missoula, Division of Biological Sciences, University of Montana, Missoula, MT 59812, USA

**Keywords:** animal flight, hummingbird, manoeuvre, inertial steering, computational fluid dynamics

## Abstract

Hummingbirds outperform other birds in terms of aerial agility at low flight speeds. To reveal the key mechanisms that enable such unparalleled agility, we reconstructed body and wing motion of hummingbird escape manoeuvres from high-speed videos; then, we performed computational fluid dynamics modelling and flight mechanics analysis, in which the time-dependent forces within each wingbeat were resolved. We found that the birds may use the inertia of their wings to achieve peak body rotational acceleration around wing reversal when the aerodynamic forces were small. The aerodynamic forces instead counteracted the reversed inertial forces at a different wingbeat phase, thereby stabilizing the body from inertial oscillations, or they could become dominant and provide additional rotational acceleration. Our results suggest such an inertial steering mechanism was present for all four hummingbird species considered, and it was used by the birds for both pitch-up and roll accelerations. The combined inertial steering and aerodynamic mechanisms made it possible for the hummingbirds to generate instantaneous body acceleration at any phase of a wingbeat, and this feature is probably the key to understanding the unique dexterity distinguishing hummingbirds from other small-size flyers that solely rely on aerodynamics for manoeuvering.

## 1. Introduction

Hummingbirds are one of nature's most remarkable flyers, capable of not only cruising flight like most other birds but also uniquely capable of sustained hovering and rapid, near omnidirectional aerobatic manoeuvres [1]. To enable these different flight modes, their flapping wings must provide the necessary forces and moments for six degrees-of-freedom (d.f.) linear and angular accelerations, in addition to maintaining weight support. In hovering flight, hummingbirds appear to have converged evolutionarily on the wing kinematics and aerodynamics of insects [2,3]. In manoeuvres, however, hummingbirds exhibited higher degree of aerial agility at low speeds, arguably, than all other birds at larger body size [4] and flying insects at similar or smaller body size [5].

**Figure 1.**
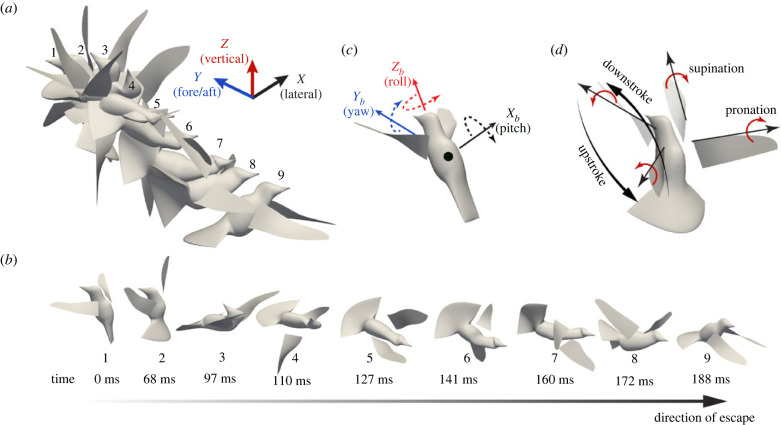
An elicited escape manoeuvre in a Rivoli's hummingbird. (*a*) Snapshots of the reconstructed escape manoeuvre in the global reference frame; (*b*) the same snapshots separated to show body orientation; (*c*) principal axes of the bird in the body-fixed coordinate system and definitions of pitch, roll, and yaw rotations; (*d*) illustration of downstroke, upstroke, pronation and supination in a cycle.

Both hummingbirds and flying insects employ unsteady aerodynamics associated with their reciprocating wing movements, including stroke acceleration/deceleration, wing pitching and dynamic changes of the angle of attack, and wing reversal [2,3,6,7]. In a hovering-to-escape manoeuvre, a Rivoli's hummingbird, for example, can evade the perceived threat, reorient its body, and accelerate toward the escaping direction in less than 0.2 second or about five or six wingbeat cycles [8,9] (figure 1). In the process, the hummingbird body undergoes rapid pitch and roll, and is capable of reaching maximum rotational speed within a single wingbeat. How are hummingbirds able to execute such a complex manoeuvre (literally) in the blink of an eye? Such rapid, within-wingbeat body acceleration cannot be achieved by insects such as fruit flies which rely on subtle deviations of wing kinematics generated by steering muscle of relatively low power capacity [10–12]. Unlike insects, hummingbirds drastically change their wing kinematics and substantially alter the aerodynamic force vector with respect to their body to generate near omnidirectional linear and rotational accelerations [8,9]. However, since their wing motion is reciprocal, the forces and torques would be reversed between half strokes and therefore could largely self-cancel. Therefore, we do not yet understand how these forces lead to the net torques needed for rapid rotational acceleration of the body. In addition, it is not clear how hummingbirds manage to control their rapid rotations without losing stability.

Altshuler *et al.* have done several studies on the manoeuverability of hummingbirds using automated video tracking of a large number of free-flight samples [13–15], and they analysed manoeuvres such as linear acceleration, simple pitch or yaw turns, as well as the complex pitch-roll turn. They found that the increased muscle capacity and the wing morphological differences (e.g. larger wing size) greatly contributed to the high manoeuverability of larger species. Combining video tracking and a quasi-steady aerodynamic model, they also studied arcing turns of hummingbirds and found that the hummingbirds control their turning velocities using body orientation but control their turning radius using asymmetrical wingbeat kinematics [16]. Despite these insightful findings, it remains unclear how the underlying mechanics enabled the sophisticated escape manoeuvre in the present study.

It is commonly accepted that hummingbirds, like other flying insects or small-size unmanned aerial vehicles (UAVs), rely on aerodynamic forces to generate manoeuvres. However, studies have shown that some animals are also adept at recruiting inertia of their limbs, bodies and tails for steering and stabilization, e.g. gecko [17] and bats [18,19]. Compared with flying insects, hummingbirds have relatively high wing-to-body mass ratio (albeit not as high as bats), and wing kinetic energy to the aerodynamic work [5], both suggesting a potentially increased role of wing inertia in manoeuverability. Previous work based on wingbeat-cycle-averaged analysis for flapping wings typically disregard the role of inertia since the cycle-averaged inertial forces sum to approximately zero [20,21]. However, the recent finding shows that hummingbirds can achieve peak rotational acceleration as fast as in one single wingbeat [8,9], suggesting that the cycle-averaged analysis may not apply to the rapid body rotations in hummingbird manoeuvres and within-wingbeat details need to be examined to understand the flight mechanics of complex manoeuvres.

To address these gaps in understanding how hummingbirds accomplish their unparalleled aerial manoeuverability, and to reveal the roles of aerodynamic and inertial mechanisms in generating within-wingbeat body accelerations, we analysed the digitized high-speed videos of elicited escape manoeuvres of four hummingbird species [8,9]. We first examined the timing of body pitch rotational acceleration within the wingbeat cycle to infer the primary sources of torque production and form our hypotheses. Then, we reconstructed the full-body kinematics of the hummingbirds from the digitized pre-labelled dots and anatomical landmarks (figure 1), and we performed high-fidelity computational fluid dynamics (CFD) and six d.f. flight mechanics simulation of the escape process. The simulation captures both spatial and temporal details of the aerodynamic and inertial force distributions from the wings, from which we calculated the total forces and torques and revealed the mechanisms that enable the hummingbirds to perform the rapid manoeuvres.

## 2. Methods

### 2.1. Summary of bird experiment and videogrammetry

Rotational accelerations of the body were extracted from previous kinematic measures of hummingbird manoeuvres in four species during elicited escape that were reported in Cheng *et al.* [8] (Rivoli's hummingbird, *Eugenes fulgens;* blue-throated mountain-gem, *Lampornis clemenciae;* black-chinned hummingbird, *Archilochus alexandri;* and broad-billed hummingbird *Cynanthus latirostris*). These data were used to perform initial analysis and develop the hypotheses on inertial and aerodynamic forces that we then tested using CFD modelling. Details of the original experimental design are in Cheng *et al.* [8]. Only a brief summary is provided here. Body mass (M) was measured using digital balance (resolution 0.01 g). The flight experiment was performed in an acrylic plastic transparent chamber with dimensions of 87 × 77 × 61 cm. The bird was fed via a 3 ml plastic syringe that was located at one side of the chamber. While it was hovering and feeding, the bird was startled from the front of the feeder by a human thrusting a black clipboard toward the bird. The bird performed the escape manoeuvre and immediately flew away from the syringe (see electronic supplementary material, movie S1 for a sample). The flight kinematics of the entire manoeuvre were recorded at 1000 frames s^−1^ with a shutter speed of 1/6000 s using three high-speed video cameras, one SA3 and two PCI-1024 (resolution 1024 × 1024 pixel) (Photron Inc., San Diego, CA, USA). The bird's body and wings were marked with 1.5 mm dots of non-toxic, water-soluble white paint prior to the experiment. A total of 18 marker points were placed, eight points on the body and five points on each wing (electronic supplementary material, figure S1*b*).

In the next step, the three-dimensional coordinates of marker points were digitized frame by frame from the videos using a custom MATLAB program [22] (electronic supplementary material, figure S1*c*). To obtain more detailed kinematics for our new analysis, five additional marker points were added: one on each wing tip, one on the trailing edge of each wing, and the fifth one on the middle of the outermost tail feather. These five points were identified based on natural marks on the feathers (electronic supplementary material, figure S1*b*).

About 200 frames were reconstructed (six to seven wingbeat cycles), which consisted of a short period of hovering, rapid pitch-up and roll, and linear accelerations. This stereotyped combination of pitch-roll manoeuvre was consistent among the bird samples across different species [8]. Thus, only two Rivoli's hummingbirds were used for the CFD study.

### 2.2. Reconstruction of the wing-body kinematics

In the bird model for CFD, each wing was represented by a zero-thickness surface whose profile was created using spline interpolation based on the digitized marker points. The interior of the wing surface was spanned by straight chordwise segments connecting the leading edge and the trailing edge. Each wing surface consisted of 721 nodal points and 1340 triangular elements. The reconstructed wing surfaces incorporated the dynamic deformation features seen in the videos including spanwise bending and twisting. A temporal spline interpolation was further introduced to smooth the transition of the nodal positions in time to facilitate the small time-step simulation. The same strategy was used previously to reconstruct hovering and cruise flight of hummingbirds [23,24].

To reconstruct the bird body, the characteristic dimensions of the bird were first extracted along the lateral and fore–aft directions within different cross sections. Based on these dimensions, a series of ellipses were used to create the cross sections from the beak to the tail, where each ellipse consisted of 80 nodes. During flight, these ellipses were transformed, including translation, rotation and deformation, to follow the nine marker points that served as the control points (one on the bill, two eyes, one on the back, two at the body-tail junction and three on the tail; see electronic supplementary material, figure S1*b*). Such transformation incorporated changes of the body orientation as well as deformations induced by the head movement and tail flaring. The bird body including head, trunk and tail was then meshed using the points on the ellipses. At certain stages, e.g. when the bird was turning its head to its left, the mesh around the neck was smoothed to avoid being overly distorted due to excessive strain. Overall, the body surface consisted of 15 836 triangular elements and 7920 nodes. A small proximal section of the wings was left out to leave a gap between the wings and body, which helps alleviate the numerical challenges caused by the situation when the two wings are in close contact with the body during supination. This gap does not significantly affect the results because during hovering or low-speed flight like the present manoeuvre, the air speed relative to the wings is small, and thus the proximal section does not generate as much force as in the scenario considered previously for cruise flight [24,25]. In addition, the proximal part has smaller distance of the moment arm than the distal part, and thus its contribution to torque is even smaller.

### 2.3. Morphological data and wing mass distribution in the model

The morphological data of the two Rivoli's hummingbirds are given in table 1. The principal axes of the body were defined as in figure 1*c* along with definitions of the pitch, yaw and roll rotations. The principal moments of inertia (MoIs) of the body without wings were taken from the estimates by Cheng *et al.* ([9], electronic supplementary material, table A1), where the same bird samples were used for their study. The off-diagonal terms of the MoIs, i.e. $I_{xy}^{b}$, $I_{yz}^{b}$ and $I_{xz}^{b}$ were small and assumed to be zero. For simplicity, the wingless body was assumed to be rigid and thus the MoIs were constant during the manoeuvre.

**Table 1.** Morphological and kinematic data of Rivoli's hummingbirds used in the CFD simulation.

parameter	Bird 1	Bird 2
mass, M	7.73 g	7.49 g
average cord length, c	1.72 cm	1.74 cm
average wing surface area, S	11.42 cm^2^	11.74 cm^2^
wing length, R	7.47 cm	7.26 cm
average wingtip velocity, V	10.97 m s^−1^ (hovering),	11.70 m s^−1^ (hovering),
	16.23 m s^−1^ (escape)	17.76 m s^−1^ (escape)
wingbeat frequency, f	24.76 Hz (hovering),	22.73 Hz (hovering),
	34.41 Hz (escape)	37.75 Hz (escape)
moment of inertia (without wings)	$I_{xx}^{b}=2,119$, $I_{yy}^{b}=2,741$, $I_{zz}^{b}=958\;g\;{\mathrm{mm}}^{2}$	$I_{xx}^{b}=2,013$, $I_{yy}^{b}=2,604$, $I_{zz}^{b}=910\;g\;{\mathrm{mm}}^{2}$

Mass distribution for the wings was obtained by scaling the experimental data from the ruby-throated hummingbird's (*Archilochus colubris*, body mass *M* = 3.245 g) wings. In the experiment [26], each hummingbird wing was cut chordwise into 11 strips, and the mass of each strip was measured using a digital scale accurate to 0.0001 g, so that the one-dimensional mass distribution was obtained from the root to the tip. Since the hummingbird's wings consist of feathers and a musculoskeletal structure of muscle and bone, we separately distributed the mass of the bony structure along the leading edge from the root to the location of the phalanges (electronic supplementary material, figure S2). The scaled mass data are in electronic supplementary material, table S1.

### 2.4. Computational fluid dynamics simulation set-up and verification

For the CFD simulation, the flow was considered viscous and incompressible, and was governed by the three-dimensional Navier–Stokes equation. The Reynolds number based on the characteristic wing-tip velocity of 30 m s^−1^ and the average chord length of 1.7 cm was set to be Re = 2550. The governing equation was discretized on a single-block, non-uniform Cartesian grid and was solved with an in-house, second-order accurate immersed-boundary method [27]. The computational domain was a rectangular box of size 24 × 21 × 24 cm, where domain translation was adopted to follow the overall shifting position of the body (the corresponding non-inertial effect due to domain translation had been incorporated in the governing equation). For the baseline simulation, a total of 294 million (700 × 600 × 700) mesh points were used. The time-step size for the simulation was Δt = 5 µs. Therefore, approximately 8000 steps were used to resolve a complete wingbeat cycle during initial hovering (or about 6000 steps for later cycles). For the simulation, a total of 1000 processor cores were used on Stampede2 at the Texas Advance Computing Center (TACC).

Mesh refinement and domain size have been verified in two separate simulations to ensure that the current mesh and domain set-up are adequate. To verify mesh convergence, a finer mesh was also used in simulation. Maximum grid resolution was employed around the wing during both cases. For baseline case, grid spacing was 0.03 cm in all three directions. On the other hand, grid spacing for finer grid was 0.02 cm in all three directions. Electronic supplementary material, figure S3 shows the comparison between baseline and refined mesh cases.

To verify the effect of domain size, the domain was increased in both sides of wingspan direction to repeat one escape wingbeat cycle simulation from *t* = 114 to 146 ms for Bird 1, where the domain was increased in the Z-direction by 5 cm (i.e. box size of 24 × 21 × 34 cm). Since the refined-mesh and the larger-domain simulations were done for an isolated wingbeat, rather than a continuous simulation from *t* = 0 (thus, potential interactions of the wings with vortices from any previous cycles were not included), an additional verification of isolated cycle simulation was also performed. All these verification results are presented in electronic supplementary material, figure S3 and show that the baseline set-up was sufficient.

### 2.5. Modelling of flight mechanics

For the simulation of the flight mechanics, the bird was considered as three separate parts, i.e. two wings and the main body, and the main body was assumed to be a rigid body going through six d.f. movement during the manoeuvre. The wing–body interaction was represented by a joint force and a joint torque. The linear acceleration of the main body can be written as

2.1 $$M_{b}\frac{dV_{b}}{dt}\;=(-F_{\mathrm{JL}}-F_{\mathrm{JR}})+M_{b}\;g\;+\sum f_{\mathrm{bi}}\;,$$

where *M*_b_ is the mass of the main body, ***V***_b_ is the linear velocity, ***g*** is the gravitational acceleration*,* $F_{\mathrm{JL}}$ and $F_{\mathrm{JR}}$ are the wing-joint force exerted on the left and right wings, respectively, and $f_{\mathrm{bi}}$ is the aerodynamic force on the body mesh nodes. Each wing-joint force was calculated by including the inertial force, gravitational force and aerodynamic force on the wing, e.g.

2.2 $$F_{\mathrm{JL}}=\sum m_{i}a_{i}-\sum m_{i}g-\sum f_{wi}\;,$$

where $m_{i}$ is mass of a mesh node on the wing, ***a***_i_ is linear acceleration of the node, and ***f***_wi_ is the aerodynamic force at the node (i.e. the difference between the aerodynamic forces on the two sides of the wing).

The MoI matrix of the main body was defined with respect to the centre of mass (CoM) as $I_{b}$, and the rotational velocity and acceleration as $\omega_{b}$ and $\alpha_{b}$, respectively. Then, the rotational dynamics of the main body can be written in the body-fixed frame as

2.3 $$I_{b}\frac{d\omega_{b}}{dt}+\omega_{b}\times (I_{b}\omega_{b})=-r_{\mathrm{OL}}\times F_{\mathrm{JL}}-r_{\mathrm{OR}}\times F_{\mathrm{JR}}-\tau_{\mathrm{JL}}-\tau_{\mathrm{JR}}+\sum r_{i}^{\prime }\times f_{\mathrm{bi}}\;.$$

Here, $\tau_{\mathrm{JL}}$ and $\tau_{\mathrm{JR}}$ are the joint torque exerted on the left and right wings, respectively, by the main body, $r_{\mathrm{OL}}$ and $r_{\mathrm{OR}}$ are the distance vectors from the CoM to the wing joints, and $\;\sum r_{i}^{\prime }\times f_{bi}$ is the aerodynamic torque on the main body ($r_{i}^{\prime }$ is the distance from the CoM to the mesh node). The wing-joint torques were calculated by including both the inertial and aerodynamic torques on the wings, e.g.

2.4 $$\tau_{JL}=-\sum r_{i}^{\prime }\times f_{wi}+\;\sum r_{i}^{\prime }\times m_{i}a_{i}\;,$$

where $r_{i}^{\prime }$ is the distance from the wing joint to the mesh node.

Once the CFD simulation was completed, the aerodynamic forces and torques on the bird's body were included in the flight mechanics simulation. That is, equations (2.1) and (2.3) were integrated in time to compute the translational velocity $V_{b}$ and the rotational velocity $\omega_{b}$.

To validate the computational model, we compared the simulated translational velocities and rotational velocities from solving equations (2.1) and (2.3) with the velocities derived from the digitized marker point positions. The results of the comparison are provided in electronic supplementary material, figure S4, along with detailed discussions. Overall, the translational velocities were well captured; however, there are significant discrepancies for the rotational velocities due to several limitations in the model that are discussed in the electronic supplementary material. In our results and conclusions, we will limit main discussion of rotational dynamics to the wingbeats whose rotational accelerations were reasonably captured by the model.

### 2.6. Power calculation

The instantaneous wing aerodynamic power $P_{A}$, wing inertial power $P_{I}$, power required for body translation $P_{T}$ and body rotation $P_{R}$ were calculated to evaluate the power consumption of the manoeuvre. Here, $P_{A}$ and $P_{I}$ were calculated by integrating over the wing surfaces the product between the force at each mesh node and the velocity $V_{i}$ as

2.5 $$P_{A}=-\sum f_{i}\cdot V_{i}\;,$$

and

2.6 $$P_{I}=\sum (m_{i}a_{i})\cdot V_{i}\;.$$

The body translational power, $P_{T}$, and body rotational power, $P_{R}$, were calculated as follows:

2.7 $$P_{T}=F_{b}\cdot V_{b}\;,$$

and

2.8 $$P_{R}=\tau_{b}\cdot \omega_{b}\;.$$

Here, $F_{b}$ is the summation of wing-joint forces from the wings to the body, and $\tau_{b}$ is the total torque acted on the body by the wings. These can be obtained as follows:

2.9 $$F_{b}=(-F_{\mathrm{JL}}-F_{\mathrm{JR}})+M_{b}g,$$

and

2.10 $$\tau_{b}=-r_{\mathrm{OL}}\times F_{\mathrm{JL}}-r_{\mathrm{OR}}\times F_{\mathrm{JR}}-\tau_{\mathrm{JL}}-\tau_{\mathrm{JR}}.$$

## 3. Results

### 3.1. Pitch-up rotation of four hummingbird species

**Figure 2.**
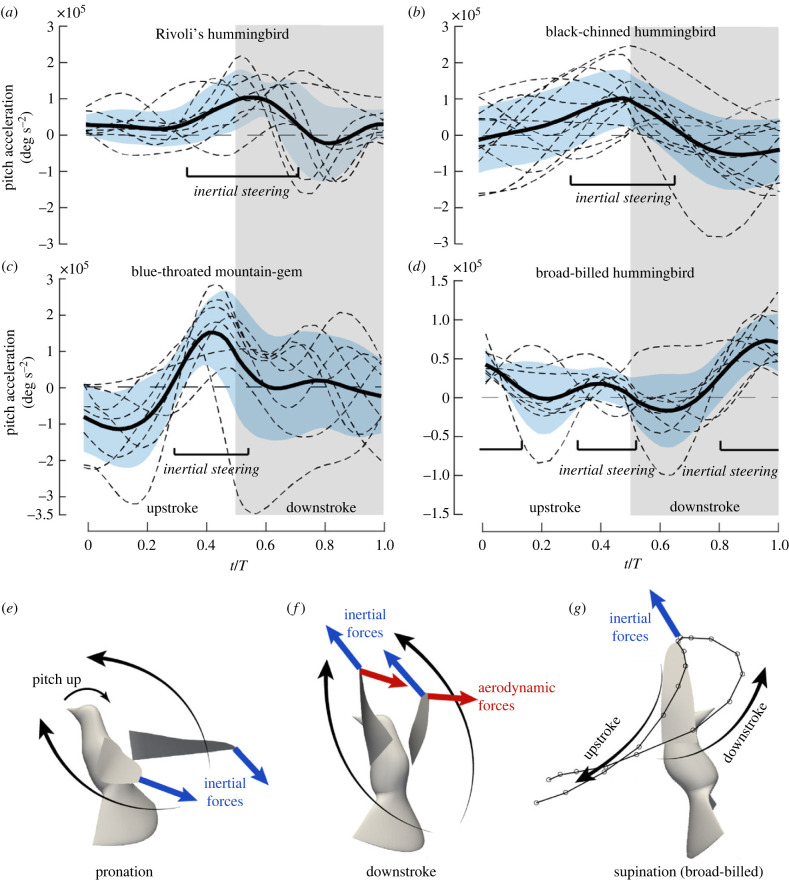
Pitch-up acceleration during the escape manoeuvre showing inertial steering. (*a*) Rivoli's (*b*) black-chinned mountain-gem, (*c*) blue-throated, and (*d*) broad-billed hummingbirds. The wingbeat period *T* was used to scale the time. Thin-dashed lines represent individual wingbeat cycles from different bird samples and trials; the thick-black line represents the average among trials; and the blue shade is standard deviation. (*e*) Illustration of inertial steering during pronation; (*f*) illustration of the aerodynamic forces counteracting the reversed wing inertial forces during downstroke; (*g*) illustration of the wing trajectory in broad-billed hummingbirds, where the inertial forces also lead to pitch-up acceleration. The forces are plotted at the wing tips for better visibility.

When hummingbirds perceived the incoming threat in the experiment, they first performed a pitch-up rotation to lean back and then rolled the body toward either left or right side. A linear backward acceleration was also initiated. To study the mechanisms of body rotation, we calculated the angular velocities and accelerations of the bird body in each flight trial for four species: Rivoli's, black-chinned, blue-throated mountain-gem and broad-billed hummingbirds. The rotational velocities included pitch, roll and yaw (figure 1*c*) and were calculated from the rotational matrix formed by the body-fixed coordinate system [28]. Since the wing inertial forces and aerodynamic forces varied at different phases, we first examined the temporal profile of the instantaneous pitch acceleration, $\alpha_{p}$ (figure 2), within the wingbeat cycles. The pitch acceleration was calculated from the time derivative of the body pitch velocity, $\omega_{p}$, so $\alpha_{p}={\dot{\omega }}_{p}$. The data were taken from the wingbeat cycles corresponding to the pitch-up motion of the individual trials (two bird samples in each of the four species, and two trials for each bird) between the start of the pitch and the start of the roll turn. To ensure consistency, the same number of cycles were taken within the same species. Specifically, three consecutive cycles were taken for black-chinned hummingbirds, and two consecutive cycles were taken for all other species. These individual cycles are shown in the electronic supplementary material, along with the pitch velocity and acceleration. All the collected data and the phase-average are shown in figure 2*a–d*, which includes the standard deviation at any time moment.

For all four species, there is a significant maximum in the pitch-up acceleration nearly concurrent with pronation when the wings reach the end of upstroke and start the next downstroke. Species-related variations are evident in figure 2. Among the four species tested, Rivoli's hummingbirds and blue-throated mountain-gems underwent the most pitch acceleration because they pitched up more than 90° so that their body became upside down in the process. Black-chinned hummingbirds pitched around 75°. Their pitching went through more significant oscillations within a cycle (e.g. the body acceleration reversed direction from pronation to supination), and pitching velocity was thus greatly reduced during downstroke. As a result, this species may need three wingbeats to achieve its maximum pitching velocity, while the other three species needed only no more than two wingbeats. Broad-billed hummingbirds only pitched up by 45°, and their angular acceleration was thus the least pronounced. One interesting feature about this species is that the pitch acceleration had more pronounced maximum around the supination than pronation.

Large variations can also be seen from plots of the individual birds and trials. In some cases, significant pitch acceleration can also appear during mid-downstroke. For flapping wings, the aerodynamic forces and the wing inertial forces generally take place at different phases. At pronation or supination when the wing stroke is reversed, the aerodynamic forces of the wings are expected to be minimal, while the wing inertial forces are probably at their maximum (figure 2*e*). On the other hand, during mid-stroke (especially downstroke since it is much more powerful than upstroke), the wings are moving at maximum velocity, and thus the aerodynamic forces are at their maximum. The specific timing of these forces could be fine-tuned by detailed kinematics such as instantaneous stroke and rotational velocities of the wings.

The observations of the data in figure 2 and the considerations of the wing forces therefore led to the hypothesis that pitch acceleration could be contributed significantly by the wing inertial forces at wing reversal, which represents an ‘inertial steering’ mechanism. At the time moment when the inertial forces were expected to be reversed, the aerodynamic forces could act against the inertial forces to maintain pitch rotation (figure 2*f*), thereby stabilizing the body from pendulum-like oscillations, although for some individual trials, the aerodynamic forces may still be the primary pitching mechanism. Thus, we further hypothesized that the combined mechanism of inertial steering and aerodynamic steering had provided the rapid and smooth body rotation during the escape manoeuvre.

The unique maximum pitch-up acceleration near supination for the broad-billed hummingbirds requires attention. At supination, the aerodynamic forces are expected to be small, and the wing inertial forces may pitch down the body. To understand this, we illustrate the wing trajectory of this species in figure 2*g*. We found that towards the end of downstroke, these hummingbirds moved their wings cranially and also backward so that the wing inertial forces around supination most likely pointed upward or even backward, thus leading to a pitch-up effect on the body. This effect can therefore also be described as inertial steering.

Guided by the hypotheses of inertial steering, we proceeded to computational modelling to examine the detailed mechanisms of torque production.

### 3.2. Aerodynamic forces and body acceleration

CFD simulation was done for the two Rivoli's hummingbirds using the reconstructed body and wing kinematics (see electronic supplementary material, movie S2 for flow visualization). Without losing generality, the results for Bird 1 are presented here for discussion; those for Bird 2 are consistent with Bird 1 and are provided in the elactronic supplementary material.

**Figure 3.**
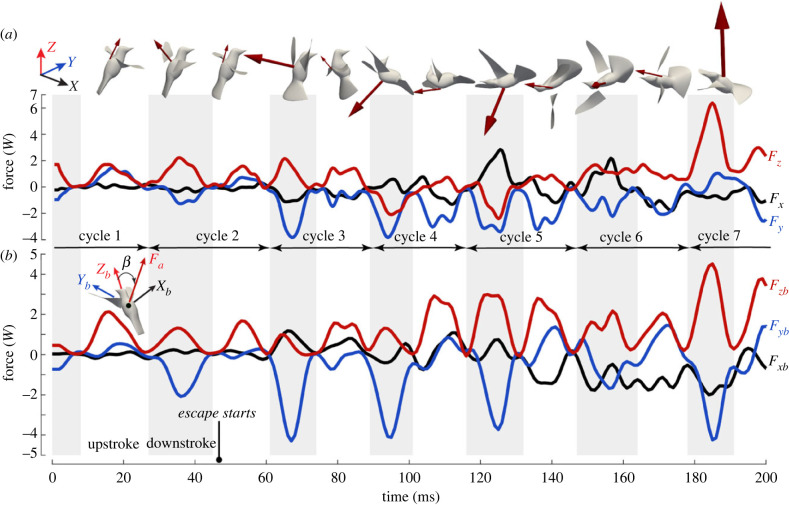
Instantaneous aerodynamic force components (normalized by body weight, *W = Mg*) on the bird (Bird 1). (*a*) In the laboratory frame and (*b*) in the body-fixed frame. The peak force vector is illustrated in insets for each half stroke.

The instantaneous aerodynamic force components changed significantly from hovering to escape (figure 3*a*; see electronic supplementary material, movie S3 for an animation). During initial hovering, the stroke plane was nearly horizontal, and the wings produced weight support in both downstroke and upstroke. Around *t* = 50 ms, the bird was startled and in response it flared the tail and started to escape. In the next two wingbeat cycles (*t* = 60 to 115 ms), the bird tilted its stroke plane cranially relative to its body. Meanwhile, the bird body was pitching backward (pitch-up) toward the direction of escape (-Y). Due to the reorientation of the stroke plane, the aerodynamic force vector in these two wingbeats was also directed backward to initiate the linear acceleration toward the escape direction. The overall force magnitude was significantly greater than in hovering (about 1.9 times) largely due to a greater wingbeat frequency and thus faster stroke speed.

In the next cycle (*t* = 115 to 145 ms), the bird continued to fly in an upside-down position, while it was also rolling toward its left side. Both downstroke and upstroke produced a large force for escape acceleration. In the process, the vertical force could become negative, and consequently the bird may temporarily lose some weight support.

In the next cycle (*t* = 145 to 180 ms), the bird continued to roll toward its left and was recovering from the upside-down position. The aerodynamic force vector rolled along with the body but overall continued to provide escape acceleration. Finally in the next cycle (after *t* = 180 ms), the bird used a powerful downstroke to stop its body from falling and to regain its altitude. The peak force reached nearly six times of body weight.

The aerodynamic force components in the body-fixed coordinate system revealed how much the aerodynamic force vectoring changed relative to the bird body (figure 3*b*). For example, during hovering the aerodynamic force vector was around $\beta =60^{\circ }$ from the Z_b_-axis at mid-downstroke and nearly zero at mid-upstroke; but during the first three escape cycles, this angle changed to around $\beta =80^{\circ }$ and $-25^{\circ }$, respectively, for mid-downstroke and mid-upstroke. Such significant changes of force vectoring allowed the bird to back up away from the oncoming threat even before its body was fully rotated.

### 3.3. Inertial and aerodynamic mechanisms of pitch acceleration

**Figure 4.**
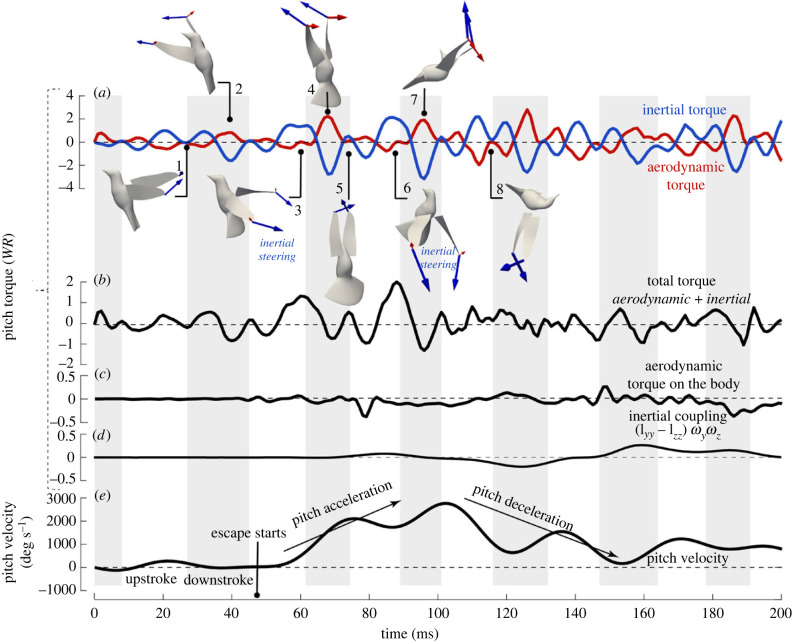
Pitching torques normalized by the body weight × wing length, *WR* (Bird 1). (*a*) The aerodynamic, inertial and (*b*) net torques produced by the wings; (*c*) aerodynamic torque on the bird body; (*d*) the body cross-product inertial term, $\omega_{b}\times I_{b}\omega_{b}$; (*e*) pitching velocity of the body directly derived from the body markers. Wing inertial (blue arrows) and aerodynamic (red arrows) forces were shown for selected time frames, where the effect of inertial steering is pointed out.

After the escape manoeuvre was initiated, both inertial and aerodynamic torques were greatly increased compared with hovering (figure 4*a*). Overall, the inertial torque had a greater magnitude than the aerodynamic torque in this case. In the next two wingbeats (*t* = 50 to 110 ms) when pitch acceleration took place, the instantaneous net pitch acceleration was largely generated by a large inertial torque around pronation when the wings decelerated toward the end of upstroke and accelerated at the beginning of downstroke. The aerodynamic torque during the rest of the stroke cycles attenuated the opposite inertial torque that was inevitably generated by reversal acceleration of the wings, which led to a stabilized pitch rotation. As a result, aerodynamic torque sustained the pitch rate within a wingbeat. These results therefore supported the hypotheses we stated earlier.

Illustrations of the wing inertial and aerodynamic forces (figure 4*a*) help us understand the torque production (see electronic supplementary material, movie S4 for a step-by-step animation in three different views). At pronation, the wings generally experienced angular deceleration from upstroke and then acceleration from downstroke; therefore, each wing produces a strong inertial force. During hovering, the two wings came close to each other behind the body at pronation (figure 4*a*, inset 1). Therefore, the inertial forces of the two wings were against each other and cancelled, and there was no significant inertial torque at pronation. However, to initiate pitch-up, the bird not only increased the wing inertial forces but also aligned them for pitch rotation. Note that the bird terminated its upstroke earlier when the two wings were still quite separated from each other at pronation (figure 4*a*, insets 3 and 6). As a result, the two inertial forces pointed backward, and they worked together to generate a large torque pitching up the body. After pitch acceleration, the wings resumed their stroke amplitude at pronation, and thus the inertial torque disappeared at pronation of later cycles. For all the wingbeat cycles, supination did not produce a similar inertial torque as pronation. This is because the two wings were close to each other at supination and their inertial forces were against each other (figure 4*a*, inset 5).

The reversed, decelerating (pitch-down) inertial torque peaked slightly after mid-downstroke (e.g. *t* = 70 and 95 ms in figure 4*a*, insets 4 and 7). At mid-downstroke, the opposing aerodynamic torque was also near its maximum and thus negated this reversed inertial effect. The aerodynamic torque was highest around mid-downstroke not only because the wing velocity was the greatest at that time, but also because the stroke plane was tilted during escape and the aerodynamic force was directed backward, thus producing an increased pitch-up torque to counteract the reversed inertial torque.

The average net pitching torque of the wings for the pitch-up acceleration stage was 0.003 N m, or 0.54 WR, where *W* is the body weight and *R* is the wing length, and this was significantly lower than the magnitude of individual inertial or aerodynamic peak torque (either could reach around 0.015 N m or 2.65 WR). This result indicates that the pitch rotation involved intricate interplay between the instantaneous aerodynamic and inertial torques, and it was largely influenced by the oscillatory nature of wing inertia. The wingbeat-averaged analyses on aerodynamic forces commonly used in flapping flight literature thus would fail to fully explain the hummingbird manoeuvrability [29,30].

The aerodynamic torque on the main body of the bird was overall much smaller in comparison with the wing torques (figure 4*c*). It mostly came from the flared tail, which created a resisting aerodynamic torque when the body pitched up. This torque may provide some pivoting effect at the tail so that the upper body could back away from the threat more quickly, and it may also provide a dampening effect in the process to help stabilize the rotation.

### 3.4. Mechanism of pitch deceleration due to inertial coupling of roll and yaw

During the pitch deceleration period (*t* = 100 to 160 ms in figure 4*a*), the wing inertial forces did not produce any significant torque at wing reversal. As explained earlier, the two wings resumed their stroke amplitude at pronation and their inertial effects cancelled each other (e.g. figure 4*a*, inset 8). The aerodynamic torque was mostly opposing the inertial torque generated during stroke, leading to small oscillations in the net torque and an extended period of balance.

Interestingly, the cross-product term of the body rolling $\left(\omega_{z}\right)$ and yawing $\left(\omega_{y}\right)$ in equation (2.3), $(I_{yy}^{b}-I_{zz}^{b})\omega_{y}\omega_{z}$, which was calculated using the directly derived angular velocities, also provided a significant pitch-deceleration torque (figure 4*d*). This effect was caused by the three d.f. rotation of a rigid body, where the body yawing and rolling would contribute to the dynamics of pitching, i.e. the angular momentum of yaw was transferred to that of pitch. Magnitude of this coupling term could reach −0.20 WR between *t* = 100 and 150 ms. Rotational velocities also show that there was significant overlapping between rolling and yawing for all the bird samples in terms of both magnitude and direction, consistent with this inertial coupling effect (quantitative analysis of all samples is deferred to a future study).

### 3.5. Mechanisms of roll acceleration

**Figure 5.**
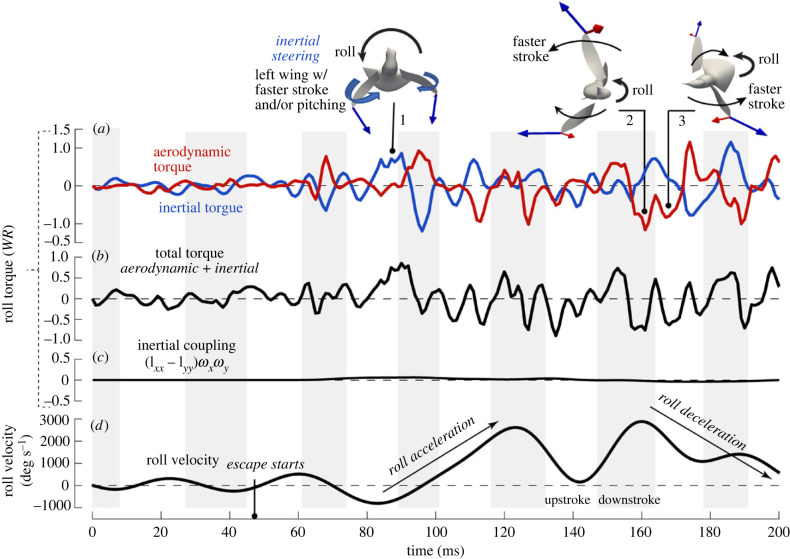
Rolling torques normalized by the body weight × wing length, *WR* (Bird 1). (*a*) The aerodynamic, inertial and (*b*) net torques produced by the wings; (*c*) the body cross-product inertial term, $\omega_{b}\times I_{b}\omega_{b}$; (*d*) rolling velocity of the body directly derived from the body markers. Wing inertial (blue arrows) and aerodynamic (red arrows) forces were shown for selected time frames. Inset 1 shows that the left wing had a greater acceleration around pronation, thus creating an inertial torque.

Both aerodynamic and inertial torques had significant contributions to roll acceleration (figure 5*a*). For both birds simulated, roll acceleration was achieved within one single wingbeat and was faster than pitch acceleration due to a lower moment of inertia for roll.

During roll acceleration from *t* = 80 to 100 ms in figure 5, the net torque was mostly contributed by the positive (leftward) inertial torque during late upstroke and pronation when the aerodynamic torque was small. In the following downstroke, the large aerodynamic torque counteracted the reversed inertial torque. This inertial steering mechanism was similar to that observed in pitch. During most of the next wingbeat (*t* = 100 to 130 ms), roll acceleration continued as seen from figure 5*d*. This period of acceleration was not well captured by the model (see electronic supplementary material, figure S4*b*), but it is likely that the same inertial mechanism was present, given very similar wing kinematics around pronation, e.g. the translational and pitch velocities of each wing, between these two consecutive wingbeats. In addition to the inertial mechanism, an aerodynamic mechanism was probably sustaining the acceleration observed in figure 5*d* during upstroke between *t* = 100 and 115 ms.

Inspection of rolling torques on the left and right wings showed that the left wing had a greater stroke speed and/or a larger pitching velocity (i.e. rotation around the wing axis). As a result, the entire surface of the left wing was moving with a higher momentum than the right wing and thus produced a greater inertial force at pronation (figure 5, inset 1). A net inertial rolling torque was thus created. After the following mid-downstroke, the inertial torque was reversed, since the left wing had to decelerate more. However, the left wing was also producing a greater aerodynamic force at this moment (electronic supplementary material, figure S5), which led to an aerodynamic torque counteracting the reversed inertial effect. The combined aerodynamic and inertial mechanisms accelerated the roll by more than 3000 deg s^−^^1^ in about 1.5 wingbeats (figure 5*d*).

### 3.6. Mechanism of roll deceleration due to aerodynamic damping

Once rolling was established, the aerodynamic torque became more negative (opposing roll) than positive, and the negative aerodynamic torque provided the dominant mechanism to stop the body from over-rotation during the roll-finish period between *t* = 155 to 180 ms in figure 5. Further inspection of the velocities and aerodynamic forces of the two wings led to conclusion that such stabilizing aerodynamic torque was largely by the passive mechanism called ‘flapping counter-torque’ [20]. Here, the flapping counter-torque was generated by combining the roll of the body and wing stroke, which led to asymmetric motion of the two wings (electronic supplementary material, figure S5). For example, during downstroke the right wing was faster than the left wing due to the left-rolling of the body (figure 5, inset 2). Similarly, during upstroke the left wing was faster than the right wing (figure 5, inset 3). The corresponding aerodynamic drag on the two wings was therefore also asymmetric and created a net torque against the body rolling. Note that roll deceleration after *t* = 180 ms was not captured in the simulation due to the model limitations discussed in the electronic supplementary material; however, it is likely that the same aerodynamic mechanism continued, given the similar asymmetric wing kinematics observed in the two consecutive wingbeats of roll deceleration.

### 3.7. Aerodynamic power of the manoeuvre

**Figure 6.**
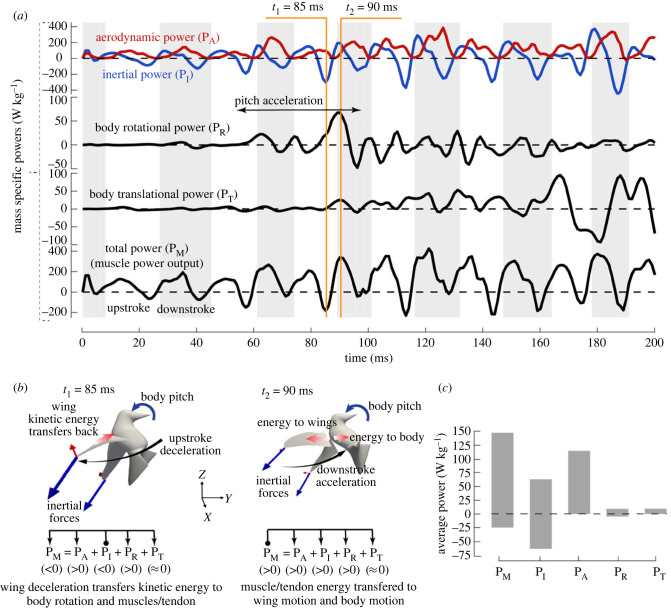
Power consumption of the escape manoeuvre. (*a*) Instantaneous mass-specific power of Bird 1, where the inertial power *P*_I_ and aerodynamic power *P*_A_ of the wings, the body rotational power *P*_R_ and translational power *P*_T_, and total muscle power output *P*_M_ were plotted. (*b*) Illustration of power flow during inertial steering. (*c*) Average positive and negative power of a wingbeat cycle throughout the manoeuvre.

The average mass-specific aerodynamic power for the entire escape manoeuvres studied here was 116.5 W kg^−1^ for Bird 1 and 124.5 W kg^−1^ for Bird 2, which was about twice of the power for hovering (46.6 and 64.3 W kg^−1^ respectively). Average mass-specific aerodynamic power for escape was 123.7 W kg^−1^ for downstroke and 105.5 W kg^−1^ for upstroke; both were approximately twice the values during hovering (64.5 and 43.5 W kg^−1^; table 2 for detailed comparison). Peak aerodynamic power during escape could be substantially higher and reach more than 350 W kg^−1^ (e.g. *t* = 126 and 186 ms, figure 6*a*). The combined inertial and aerodynamic power, which represents the majority of total muscle power output, may reach around 400 W kg^−1^ during downstroke for both birds.

**Table 2.** Average mass specific aerodynamic power (W kg^−1^) during hovering and escape cycles for both birds used in the CFD simulation.

	hovering (W kg^−1^)			escape (W kg^−1^)		
	downstroke	upstroke	whole cycle	downstroke	upstroke	whole cycle
Bird 1	55.7	38.9	46.6	126.6	96.8	116.5
Bird 2	73.3	48.2	64.3	120.8	114.3	124.5
average	64.5	43.5	55.5	123.7	105.5	120.5

The total power output from the muscles at any time moment, *P*_M_, was split among the wing inertial power, *P*_I_, the wing aerodynamic power, *P*_A_, power for the body's rotation, *P*_R_, and power for the body's translation, *P*_T_ (figure 6*a*), so that *P*_M_ = *P*_I_ + *P*_A_ + *P*_R_ + *P*_T_. For flapping wings in general, the wing inertial power is positive when the wings are accelerating and thus gaining kinetic energy; conversely, the wing inertial power is negative when the wings are decelerating and releasing the kinetic energy. All or part of this negative inertial power is converted to the aerodynamic power if the wings move against significant aerodynamic forces at the time; thus, the muscle outpower is reduced. The remaining negative power, if any, could potentially be stored in the wing muscle system [31,32]. Overall, the wing inertial power sums up to zero over a wingbeat.

During escape, the magnitude of the wing inertial power was significantly increased as compared with that during hovering, and this included both positive and negative inertial power (figure 6*a*). However, during downstroke most of the negative wing inertial power was transferred to the aerodynamic power, and the muscle output power was mostly positive. This feature may be beneficial since the negative energy may not be all stored by the muscular system [33].

Significant negative total power still existed toward the end of upstroke since the aerodynamic power was not high enough to absorb the negative wing inertial power. Yet, by transferring the majority of the negative inertial power to aerodynamic power, the cycle-averaged positive total wing actuation power is only moderately higher than the aerodynamic power (figure 6*c*). In comparison with the wing inertial or aerodynamic power, the body rotation and translation powers were much lower (figure 6*a* and *c*). Furthermore, the body rotation only required power input temporarily during the rotational acceleration. The sources of rotational power could come either from the negative inertial power, or from the muscle output, as explained below.

For example, at *t*_1_ marked out in figure 6*a* (*t* = 85 ms) when inertial steering was happening, the wings were decelerating toward the end of upstroke (figure 6*b*), and the body was pitched up due to the wing inertial forces as discussed earlier. As a result, the negative wing inertial power due to wing deceleration (*P*_I_ < 0) was partially transferred to pitch rotation of the body in addition to transferring back to muscles (i.e. *P*_R_ > 0 and *P*_M_ < 0). At *t*_2_ (*t* = 90 ms) when the wings were starting downstroke, the inertial power was positive (*P*_I_ > 0) due to wing acceleration. The reaction forces at the shoulder joints continued to pitch up the body. Thus, the muscle power at this time was output to simultaneously power wing acceleration and body pitching (figure 6*b*) with *P*_M_, *P*_I_, *P*_R_ all being positive. These results indicate that the bird's inertial steering, which was enabled by the angular momentum transfer from the wings to the body, as discussed in the torque analysis, was also partially supported by the energy transfer from the wings to the body.

## 4. Further discussion

As pointed out by Cheng *et al.* [8], hummingbirds change wing kinematics significantly relative to their body from hovering to the escape manoeuvre, which leads to corresponding large alterations of aerodynamic force vectoring with respect to their body axes. In addition to confirming this result through computational fluid dynamics simulation of the detailed aerodynamics, the present study has provided novel insight about how the hummingbirds used a combination of the inertial and aerodynamic forces associated with reciprocating wing strokes to generate the force moments for fast and well-controlled body rotation. Contrary to the perhaps intuitive thought that the aerodynamic forces create the torques needed to drive the pitch, roll and yaw rotation of the body during a manoeuvring flight, our results revealed that the torques for driving the pitch and roll acceleration in an escape manoeuvre could also come from the wings' inertia forces in addition to the wings’ aerodynamic forces; the aerodynamic torques may provide the counteracting effect against the opposite inertial torques due to reversal of the wing stroke. In such cases, the wing stroke responsible for pitch and roll acceleration could be roughly divided into two different phases. One was an ‘inertial-steering phase’, which was around wing reversal (pronation or supination). In this phase, the inertial torques from the wings drove the body's rotational acceleration. The second was an ‘aerodynamic phase’, which included mostly mid-stroke. In this phase, the aerodynamic torques counteracted the reversed wing inertial torques and helped maintain the pitch and roll velocities. With these two phases, the hummingbirds could achieve improved agility for rapid rotations.

The high magnitude of the wing inertial forces could provide short burst moments needed for fast body rotational acceleration. For example, the inertial rolling torque from the wings was around 0.42 WR during roll acceleration. Given that the roll moment of inertia of the body is about $I_{zz}^{b}=0.022\;MR^{2}$, the burst torque is capable of generating a roll velocity of more than 2000 deg s^−^^1^ within 15 ms. For pitch acceleration by wing pronation, the inertial torque was around 0.88 WR, which may accelerate pitch to 2000 deg s^−^^1^ within 15 ms for the pitch moment of inertia at $I_{xx}^{b}=0.05\;MR^{2}$. The wing inertia thus may generate a significant inertial steering mechanism for the hummingbirds to achieve rotational agility.

Inertial steering is also used by some other animals for manoeuvering and stability in aerial actions. For example, a falling gecko relies on its tail to perform rapid, zero-angular momentum air-righting and turn the body [17]. The hawkmoth moves its abdomen dynamically to pitch the thorax and thus redirects lift forces for effective flight control [34]. The rose-breasted cockatoo changes the moment of inertia of its wings through wing flexing to generate net inertial torque in a stroke cycle for a roll reorientation in a flapping, banked turn [35]. Likewise, bats also adjust the shape and moment of inertia of their high-mass, highly deformable wings to execute complex aerial rotations [18]. However, unlike those animals that use the inertia of other body parts like tail or abdomen, the hummingbirds produced inertial steering through their wings while they were in flapping motion. In addition, unlike cockatoos and bats, the hummingbirds did not change the wings' moment of inertia to generate the net inertial torque, which would require drastic changes of the wing shape. While using inertial steering, the hummingbirds instead took advantage of the favourable inertial torque within one wingbeat and cancelled out the unfavourable inertial torque by synchronizing it with the favourable aerodynamic torque.

Flying insects, as well as small UAVs, typically utilize aerodynamic mechanisms for flight manoeuvres. For example, fruit flies employ subtle deviations from hovering wing kinematics [10–12] to alter lift and drag and generate steering force moments for body rotations in saccade. We interpret that this is due, at least in part, to the low inertia of their body and limitation of their sensorimotor system, as accelerations generated by large deviations would destabilize their flight and their sensorimotor system may not respond rapidly enough [36]. Fixed-wing UAVs use elevators, rudders and ailerons to generate aerodynamic moments for pitch, yaw and roll controls. Multi-rotor drones manoeuvre by adjusting the aerodynamic lift of rotors. The novel use of inertial steering for flight manoeuvres by the hummingbirds could therefore be an inspiration for those seeking to develop highly manoeuvrable aerial vehicles.

## Data Availability

Supporting data can be found from the Harvard Dataverse: https://doi.org/10.7910/DVN/B9B1VD [37]. The data are provided in electronic supplementary material [38].
